# Epidemiological inference at the threshold of data availability: an influenza A(H1N2)v spillover event in the United Kingdom

**DOI:** 10.1098/rsif.2024.0168

**Published:** 2024-08-07

**Authors:** John A. Fozard, Emma C. Thomson, Christopher J. R. Illingworth

**Affiliations:** ^1^ MRC-University of Glasgow Centre for Virus Research, Glasgow, UK; ^2^ Department of Clinical Research, London School of Hygiene and Tropical Medicine, London, UK

**Keywords:** spillover events, epidemiology, rejection sampling, A(H1N2)v influenza

## Abstract

Viruses that infect animals regularly spill over into the human population, but individual events may lead to anything from a single case to a novel pandemic. Rapidly gaining an understanding of a spillover event is critical to calibrating a public health response. We here propose a novel method, using likelihood-free rejection sampling, to evaluate the properties of an outbreak of swine-origin influenza A(H1N2)v in the United Kingdom, detected in November 2023. From the limited data available, we generate historical estimates of the probability that the outbreak had died out in the days following the detection of the first case. Our method suggests that the outbreak could have been said to be over with 95% certainty between 19 and 29 days after the first case was detected, depending upon the probability of a case being detected. We further estimate the number of undetected cases conditional upon the outbreak still being live, the epidemiological parameter *R*_0_, and the date on which the spillover event itself occurred. Our method requires minimal data to be effective. While our calculations were performed after the event, the real-time application of our method has potential value for public health responses to cases of emerging viral infection.

## 1. Introduction

Viral transmission from animals to humans poses a serious threat in terms of its potential to generate novel pandemics [1]. Influenza viruses have a track record of causing serious impacts on human health, with the 1918, 1957 and 1968 pandemics each being associated with more than one million deaths [2,3].

While such pandemics are rare events, they occur in a context of much more frequent animal-to-human spillover events: in 2022, epidemiological monitoring detected close to 60 cases of avian and swine influenza infection in humans worldwide [4]. While most of these events do not lead to large numbers of people being infected, at the very earliest stages of detection the distinction between an outbreak that will remain localized, and an outbreak that will go on to seed a pandemic, may be small. Efforts are required to understand spillover events in their earliest stages.

Statistical approaches for understanding outbreaks work on very different quantities of data [5]. The SARS-CoV-2 pandemic saw the implementation of a broad range of computational and statistical approaches to track the nature and impact of the virus. Studies early in the pandemic characterized the epidemiological properties of the virus [6,7] and produced estimates of the epidemiological reproductive number *R*_0_ in different contexts [8]. Methods for ‘nowcasting’ combined multiple datasets to estimate local levels of viral prevalence [9,10]. A UK-wide project generated and shared hundreds of thousands of SARS-CoV-2 viral sequences [11]. Genome sequence data were used to study virus evolution and transmission on multiple scales [12–16].

Soon after a spillover occurs, data limitations may present themselves. Where multiple instances of infection from an emerging pathogen are observed, epidemiological models can highlight possible viral adaptation [17]. Inferences can be made of the epidemiological transmission parameter *R*_0_ [18] and of differences in *R*_0_ across settings [19]. Sequence data can be used to assess the evolutionary origins and epidemiological characteristics of emergent viruses [20,21].

At the very earliest stages of an outbreak, data limitations may be severe; the detection of a spillover event may begin with a single case of infection. To explore what might be achieved in this minimal case, we here investigate a case of human influenza A(H1N2)v virus infection, observed in the UK in November 2023. The detected case had no known contact with pigs, so was assumed not to be the first case in the outbreak [22]. After the one detection, no further observations of infection were made. Considering the period immediately following the detection, we use a novel method to estimate the probability of the outbreak having ceased, and the potential number of undetected cases of infection. We discuss the potential for minimal datasets to inform the public health response in the days following the detection of a viral spillover event.

## 2. Results

As a precursor to our method, we estimated the probability of a single case of influenza A(H1N2)v being detected as being between 4 and 10%. Publicly available data show that in week 48 of 2023, the UK rate of GP consultations for influenza-like illness (ILI) was 4.6 per 100 000 individuals, equal to a national total of approximately 3100 consultations per week. Following these consultations, 557 samples were tested as a part of a sentinel swabbing scheme, suggesting that approximately 18% of GP consultations for ILI led to swabbing and further testing [23]. Following the detection of a case, swabbing was increased in the Yorkshire and Humber regions, where the case was found [23]. While data specific to the UK population are sparse, published estimates suggest that between 25 and 50% of individuals with ILI might seek healthcare [24,25]. Our estimate range was derived from these values.

**Figure 1.**
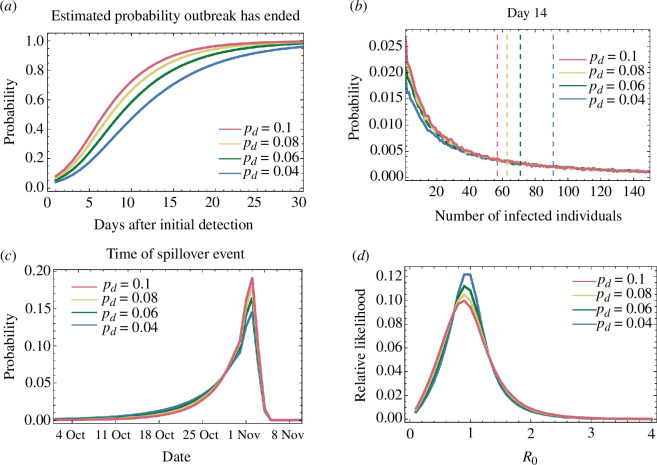
Statistics describing the influenza A/H1N2 spillover event calculated using our bootstrapping method. (*a*) Time-dependent probability that the outbreak had died out. The value *p*_d_ describes the probability of a case of A/H1N2 influenza being detected. (*b*) Calculated distribution of the number of infected individuals 14 days after the date of the first detection, conditional on the outbreak having not died out. The vertical dashed lines show the median values of each distribution. (*c*) Calculated distribution of the time at which the primary case was infected. The detection of the first case was on 23 November. (*d*) Estimate of the epidemiological parameter *R*_0_ for the influenza A/H1N2 virus involved in this spillover event.

We evaluated the properties of the A(H1N2)v influenza outbreak using likelihood-free rejection sampling [26], first carrying out a form of historical nowcasting. The outbreak involved a single detected case, which was believed not to be the primary case of infection. We generated large numbers of simulated influenza outbreaks. Each simulation was characterized by a proposed value *R*_0_, with cases of infection being ‘detected’ with fixed probability, as mentioned above. Given a day following the detection of the outbreak, we identified those simulations that matched the observed data up until that day, in so far as only a single case was detected. Examining these simulations we estimated that, 14 days after the first detection of A(H1N2)v influenza, the probability that the outbreak had died out was between 66 and 88% (figure 1*a*). The range in this value reflects uncertainty in the probability of a case being detected, with higher detection probabilities leading to greater probabilities of the outbreak having ended. At a detection rate of 4%, we inferred that the outbreak could be said to have ended with 95% confidence by day 29 after the first detection; the same conclusion could be drawn by day 19 given a detection rate of 10%.

We further estimated the number of active cases of infection, conditional upon the outbreak having not died out. In the considered case, the median number of active cases 14 days after the detection of the first case was estimated as between 57 and 91 (figure 1*b*). Estimated numbers of active cases conditional on the outbreak remaining active decreased slowly with time, considering days successively further from that of the detection (electronic supplementary material, figure S1). Our model, therefore, suggested a dichotomy of potential circumstances: the outbreak was likely to have died out but could have involved many active cases if it was still alive. We note that, soon after the detection of the first case, there exists the potential for the outbreak to involve very large numbers of cases. This result is explained by the 18 day delay between symptom onset and detection for the first detected case [27]. Given a high *R*_0_, this delay would have been sufficient for the virus to have spread substantially within the population. A 95% worst-case scenario incorporating the possibility of the cessation of the outbreak showed a rapid drop in cases to zero between 15 and 30 days after the detection of the first case (electronic supplementary material, figure S2). More rapid identification of outbreaks limits the potential for spread prior to detection.

We made a retrospective estimate of the time of the first, undetected, case of infection; estimates were made on a nominal date 90 days after the first detection. Our model predicted that this first infection occurred between 25 and 28 days prior to the first detection, between 26 and 29 October dependent on the detection rate, only a few days before the first detected case became symptomatic (figure 1*c*). The inferred likelihood function is steeply skewed, with a 95% confidence interval across detection rates between 6 October and 3 November. However, the interval between the time of the first infection and the time at which the first detected individual became symptomatic was likely short, with the detected individual potentially being a direct contact of the index case.

We also made a retrospective estimate of *R*_0_ for this outbreak, obtaining a value of 0.9 (figure 1*d*). This lies below the threshold necessary to sustain an outbreak and is lower than estimates for seasonal influenza viruses, for which *R*_0_ is in the region of 1.3 [28]. The very sparse data used in making our estimate led to a large confidence interval for this estimate, between 0.1 and 2.3 across detection rates; further data would sharpen the inferred distribution.

Exploring our data further, we examined the extent to which our retrospective estimates of *R*_0_ and the time of first infection could have been made closer to the observation of the first case. In the first few days after the detection of the case, there was little power to rule out large values of *R*_0_ (electronic supplementary material, figure S3*a*), but large values were progressively excluded with time. In contrast, the inferred time of the first case of infection was relatively stable, with estimates calculated a few days after the observation being very close to our final estimate (electronic supplementary material, figure S3*b*).

Our results are dependent upon the prior distribution chosen for *R*_0_. By default, we used a uniform prior between 0 and 4, but the highest values in this range are beyond those previously observed even for the 1918 pandemic virus [29]. Recalculating results with a uniform prior between 0 and 2 did not strongly affect our results (electronic supplementary material, figure S4).

## 3. Discussion

Examining data describing a spillover event of a swine influenza virus into the human population, we used a method of rejection sampling to explore what can be learnt, both at the time of the immediate response and, in retrospect, from the limited public data describing this event. Our method provided time-dependent estimates of the probability that the outbreak had died out, and for the number of undetected cases in the case that the outbreak was still live. It further provided estimates for the date of the spillover event itself and for the parameter *R*_0_. Our model achieves what it does because both the observation of a case of infection and the subsequent non-observation of cases are informative for the model: the failure to observe a second case of infection is an important piece of data. Over time, as more cases are not observed, this makes a rapidly growing outbreak less likely.

Our work pushes at the boundary of the amount of data required to learn about a spillover event, showing that even minimal data are sufficient to draw early and provisional conclusions about the outbreak. Our approach may be of value in a public health context: estimating the certainty with which we can say whether an outbreak is likely to have died out could inform decisions about the investigation of cases and the resources applied to this task. Simultaneously, the provisional nature of our conclusions should not be underemphasized. Methods such as contact tracing would provide substantially more information about the potential for the virus to have spread, while genome sequencing of any further cases would facilitate phylogenetic and other genomic approaches to epidemiology.

Our approach is limited by its simplicity. For example, we assumed a homogeneous population, in which each infected person is equally infectious to the others, and neglected the potential for evolution to change the infectivity of the virus. We modelled data collection in a simple manner, assuming, for example, a fixed time between symptom onset and test result. We note that the epidemiological dynamics of infection, expressed as distributions of the time to symptom onset and to infecting others, are of critical importance to our method, but were of necessity based upon distributions inferred for other influenza strains: the A/H1N2 virus detected potentially would not mirror seasonal influenza in this way. Many of the assumptions made by our method could be elaborated upon, for example, by allowing for heterogeneity in transmission [30]. The limited data available in this case did not encourage the use of more complex models.

The variation in our results under different detection scenarios highlights the potential value of improved systems for detecting cases of infection following a spillover event. In this case, detection efforts were stepped up in the region of the detection of the first case. More thorough and faster testing provides a greater certainty that a spillover has not led to a persistent outbreak and reduces the potential for an outbreak to grow undetected.

While we have applied our model to a specific event, describing the spillover of influenza A(H1N2)v into the human population in the UK, our approach has the potential for broader use. Given reasonable estimates for epidemiological parameters, viruses other than influenza could also be modelled. Events involving more than one detection of a positive case could also be assessed, though as the number of cases of infection increases our approach becomes less computationally efficient. Once large amounts of data become available, alternative methods for epidemiological inference are likely to perform better than our own. Our approach is of potential value in the first stages of an outbreak when data are most limited.

## 4. Methods

We used likelihood-free rejection sampling to evaluate the likely state of the outbreak underlying the available data [31]. This method generated a large number of simulated outbreaks, retaining only those which were compatible with the data collected from the A(H1N2)v outbreak. We denote by *t*_o_ the number of days since the day on which the first detected case was observed. For the historical nowcasting calculations, simulations that exactly matched the data up to time *t*_o_, with a single detected case, were accepted. Different values of *t*_o_ were assessed; where retrospective estimates of parameters were made, these were calculated at *t*_o_ = 90 days.

### 4.1. Simulation of outbreaks

Time was modelled discretely, in units of whole days. Each simulated outbreak started with a primary case. The simulation then proceeded day by day. Infected individuals were assumed to become symptomatic a random number of days *t*_s_ after being infected. Every symptomatic individual infected a total of *R* others, where *R* was Poisson distributed with parameter *R*_0_. The time of each infection event occurred a random number of days *t*_i_ after symptom onset. The random numbers *t*_s_ and *t*_i_ were drawn from Weibull distributions with parameters representing influenza infection that were described in a previous publication [32] and rounded to the nearest integer, where *w* is the cumulative distribution function


$$w\left(x,a,b\right)=1-e^{-{\left(x/b\right)}^{a}}.$$

*t*_s_ had probability mass function


$$P\left(t_{s}=t\right)=w\left(t_{s}+\frac{1}{2},a,b\right)-w\left(\max\left\{t_{s}-\frac{1}{2},0\right\},a,b\right),$$

with a similar expression describing *t*_i_. Parameters for the distribution of *t*_s_ were *a* = 7.4026 and *b* = 1.7375, while parameters for the distribution of *t*_i_ were *a* = 1.0314 and *b* = 1.0025.

We modelled the detection or non-detection of each case in our simulation, detection occurring with fixed probability *p*_d_. Detection was assumed to occur 18 days after the day of symptom onset, following data from the influenza A(H1N2)v case [27]. An exception was made for the primary case in the outbreak, which was modelled not to be detected on the basis that the index case had no contact with animals [22]. Altering this 18 day delay to a uniformly distributed parameter in the interval 14–22 days led to a broader peak for the estimated time of infection of the primary case (electronic supplementary material, figure S5). Modelling an increase in the detection probability *p*_d_ to 0.2 for cases that became symptomatic at or after the detection of the index case led to a faster convergence in the probability the outbreak had ended (electronic supplementary material, figure S6). To implement this, the higher detection probability was used for cases whose detection time was 18 days or more after the first detected case.

Each outbreak was simulated either until it died out, with no more cases of infection existing, or until the first day we were certain whether the number of cases detected in the simulation matched the number of detected cases up to the observation time.

We assumed a uniform prior over the epidemiological parameter *R*_0_ within the window [0.1, 4.0]. A grid-based search was performed, conducting 10^6^ simulations for each of 40 equally spaced discrete values of *R*_0_ from 0.1 to 4.0.

Once simulations were complete, we calculated the proportion of simulations for each *R*_0_ that were accepted, $P_{t_{o}}\left(\text{accepted}|R_{0}\right)$ . Normalizing this statistic gave the posterior probability of *R*_0_ at a given observation time *t*_o_


$$P_{t_{o}}\left(R_{0}|\text{accepted}\right)=\frac{P_{t_{o}}\left(\text{accepted}|R_{0}\right)\pi \left(R_{0}\right)}{\sum_{R_{0}}P_{t_{o}}\left(\text{accepted}|R_{0}\right)\pi \left(R_{0}\right)},$$

where the prior $\pi \left(R_{0}\right)$ is uniform. Using the accepted simulations, we estimated properties of the viral population, using the formula


$$P\left(Q|\text{accepted at }t_{o}\right)=\frac{\#\left(\text{simulations accepted at }t_{0}\;\text{and}\;Q\right)\;}{\#\left(\text{simulations accepted at }t_{0}\right)},$$

for properties *Q* including the number of infected individuals being *k* at time *t* days after the detected case, the outbreak having died out at time *t* days after the detected case or the time of the first infection having occurred a specific number of days before the detected case.

We note that alternative prior distributions for *R*_0_ could be used in our calculation; we show in the electronic supplementary material the results of reducing the upper bound on this. In the calculations above, we assumed that infection lasted for 7 days following infection. Altering this to a uniformly distributed random length of infection between 5 and 9 days made little difference to our results (electronic supplementary material, figure S7).

## Data Availability

All data were obtained from publicly available sources. Our method is named OINK (Outbreak Inference given Negligible Knowledge) and is available via Zenodo [33]. Supplementary material is available online [34].
